# Dual-mode switchable and reconfigurable Van der Waals phototransistor for multi-state image encryption

**DOI:** 10.1038/s41377-026-02358-7

**Published:** 2026-07-01

**Authors:** Yuanfang Yu, Senyao Tang, Nanjie Jiang, Yuwei Zhang, Xiaorui Jin, Jiaxin Gong, Huijuan Zhao, Anran Wang, Dongyang Wan, Zhenhua Ni, Xinran Wang, Li Gao

**Affiliations:** 1 State Key Laboratory of Flexible Electronics (LoFE) & Institute of Advanced Materials (IAM), School of Materials Science and Engineering, Nanjing University of Posts and Telecommunications, Nanjing, China; 2 School of Physics and Key Laboratory of Quantum Materials and Devices of Ministry of Education, Southeast University, Nanjing, China; 3 School of Science, Jiangsu Key Laboratory of Quantum Computing Science and Devices, Nanjing University of Posts and Telecommunications, Nanjing, China; 4 School of Integrated Circuits, Nanjing University, Suzhou, China

**Keywords:** Electronics, photonics and device physics, Imaging and sensing

## Abstract

Switchable phototransistors equipped with high-sensitivity, dynamic encryption, low-power consumption, and CMOS processing compatibility are key components for optoelectronic encryption chips. Two-dimensional van der Waals (vdWs) heterostructures provide a solution toward this goal, despite continued efforts, excessive dependence on gate voltage modulation, multi-wavelength excitation and polarization modulation causes unsolved issues of high-power operation, channel interference and limited integration level, respectively. Here, we demonstrate a dual-mode vdWs phototransistor based on PtTe_2_/WS_2_ heterostructure. Through bias-modulated band alignment and carrier dynamics, the operation mechanism can be switched between photoconductive and photovoltaic modes. In photoconductive mode, the prolonged carrier lifetime donates a large photogain, yielding a high responsivity of 1.37 A W^−1^. In photovoltaic mode, a wide unilateral depletion region effectively suppresses the dark current, contributing outstanding specific detectivity of 9.42 × 10^14^ Jones, weak light detection capability (~pW level), and high-speed response (rise time ~26.3 μs, fall time ~22.6 μs). More importantly, high on/off ratio of ~10^5^ and four distinct current states have been achieved via dual-mode operation in the phototransistor, enabling the realization of multiple optical logic gates (XNOR, NOR and XOR) and multi-state quaternary image encryption with superior average correlation coefficient of adjacent pixels of 0.03. This reconfigurable device provides a versatile platform for constructing multi-functional photoelectronic chips and advancing secure optical communication technologies.

## Introduction

The advent of the Big Data era has led to an exponential increase in the volume of information transmitted^1–3^, thereby imposing more stringent requirements on guaranteeing data security^4–6^. By directly encrypting optical signals during transmission, modern optical communication encryption technology is of paramount significance, particularly in the fields of the Internet of Things, data centers, and cloud computing, where secure information transmission is critically required^7–9^. Currently, traditional encryption devices for optical communication based on silicon still face the bottleneck in balancing high sensitivity^10^, dynamic encryption^11^, and low power consumption^12^. Binary-state encryption obtained by silicon-based photoelectric modulators lacks dynamic modulation diversity and adaptive key mechanisms, making it more susceptible to interception and analysis by adversaries^9^. Therefore, an encryption device capable of multi-state modulation is of great importance for securing optical data transmission.

Fortunately, two-dimensional (2D) van der Waals (vdWs) heterostructure devices are expected to provide a feasible solution of achieving multi-state encryption. Their high carrier mobility, electrical tunability^13^ support the dynamic encryption of optical signals^14^, while the weak vdWs force at the interface is conducive to heterogeneous integration with Complementary Metal Oxide Semiconductor (CMOS) circuits^15,16^. In addition, dark current in 2D phototransistor can be effectively suppressed through band alignment engineering^17^. This is beneficial for increasing the on/off ratio and improving the signal-to-noise ratio (SNR). Thus, 2D phototransistor provides a physical basis for breaking through the bottleneck of traditional devices and achieving high-speed and high-density integration of dynamic key generation and anti-jamming optical networks.

To meet the dynamic defense requirements in optical communication encryption systems and achieve logic encryption^18,19^, several approaches for photocurrent modulation in 2D devices have been developed, such as gate voltage modulation^20^, polarization sensitivity^21^ and multi-wavelength excitation^22^. However, gate voltage modulation requires high power consumption and a complex external circuit. Polarization modulation requires extra optical components, e.g., splitters, which expands system’s volume and hinder on-chip integration. In addition, multi-wavelength excitation requires multiple light sources, thereby decreasing resistance to environmental disturbances and increasing vulnerability to channel interference. Beyond these 2D modulation schemes, many organic dual-mode or multi-state photodetectors achieve current state diversity through stacked photoactive sub-devices^23^, interface-layer engineering to alternate between photovoltaic and photomultiplication modes^24^, or bias voltage-dependent spectral selectivity^25^ in multi-absorber architectures. These designs often require higher operating bias voltage or additional interfacial suppression to mitigate dark current. So far, developing vdWs devices with switchable response and multi-state encryption ability, and at the same time, satisfying the requirements of high-sensitivity, low-power consumption, and on-chip integration is still a great challenge.

In this work, we present a PtTe_2_/WS_2_ 2D vdWs heterostructure phototransistor with dual-mode operation. The vdWs heterostructure and metal electrodes are completely vdWs integrated by the dry transfer technique. Interfacial defects caused by direct metal deposition and surface Fermi pinning effect are prevented, ensuring effective electrical modulation and dynamic photoelectric response. The width of the unilateral depletion region at the heterostructure can be adjusted by the bias voltage (*V*_ds_), facilitating a switchable mechanism between photoconductive (PC) and photovoltaic (PV) modes. In PC mode, the unilateral depletion region is sufficiently narrow and defect trapping of carriers is dominant. The prolonged lifetime of carriers leads to a high photo-conductive gain and high responsivity (1.37 A W^−1^). In PV mode, the wide unilateral depletion region with large energy-band bending effectively suppresses dark current and accelerates the separation of photocarriers. The specific detectivity (D^*^) of the device is as high as 9.42 × 10^14^ Jones with a high-speed photoresponse (rise time ~26.3 μs, fall time ~22.6 μs). The versatility of the device is demonstrated in high-resolution imaging, opto-logic gate encryption (including XNOR, NOR and XOR). The average correlation coefficient of adjacent pixels of four-state current encryption images is reduced to 0.03, representing highly efficient image encryption performance. This work not only provides a viable solution for CMOS-compatible secure optical communications, but also the development of multifunctional vdW optoelectronics.

## Results

### Dual-mode switchable photoresponse via bias modulation

**Fig. 1:**
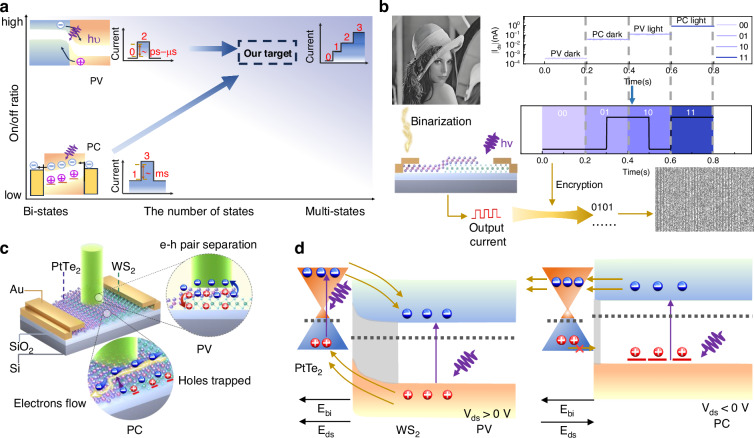
Schematic diagram of PtTe_2_/WS_2_ dual-mode switchable vdWs device. **a** Description of the trade-off between the on/off ratio and the working states of the 2D material vdWs device. The upper left band diagram represents the PV mode, and the lower left is the PC mode. **b** These four current levels that can be generated by dual-mode switching correspond to the defined quaternary numbers. 0–3 respectively represent dark current at PV mode; dark current at PC mode; source-drain current under light illumination at PV mode; source-drain current under light illumination at PC mode. **c** Schematic illustration of the proposed PtTe_2_/WS_2_ dual mode switchable vdWs device under light illumination. **d** Band diagram and carrier transport at bias voltage of 3 (left panel) and −3 V (right panel), respectively. *E*_bi_ represents the built-in electric field, and *E*_ds_ represents the voltage between the source and the drain, that is, *V*_ds_. The grey area represents the depleted region; the yellow line represents the carrier transport direction

An ideal encryption device needs to achieve a high on/off ratio characteristics and easily discernable multi-state current response under small external electric fields. Figure 1a shows the general on/off ratio characteristics and binary operation mechanism of 2D phototransistors. For typical PC devices, although the two-state current can be obtained through bias voltage modulation, their on/off ratio is generally small. On the other hand, PV devices can exhibit a high on/off ratio, but their photocurrent is usually extremely low, typically on the order of several hundred nA in 2D vdWs devices^26^. This brings considerable challenges to current discrimination and reading^27^. In addition, it is quite challenging to obtain four distinguishable current states in a single device, while multi-level current operation is our target.

By modulating the bias voltage in 2D heterostructure devices, tunable band structure engineering and carrier dynamics modulation can be achieved, enabling swift switching between PV and PC modes. This strategy not only integrates the respective advantages of both modes for information encryption applications, but also facilitates multi-level current switching with an appropriate on/off ratio. The four working states obtained by light intensity and bias voltage modulation can be identified and defined by the corresponding quaternary number (Fig. 1b). Specifically, by adjusting the on/off state of the laser and the positive/negative state of the bias voltage, four different levels of current can be obtained. These levels are defined in ascending order of current magnitude as 0, 1, 2 and 3. On this basis, the original image is first encoded, and then the output current is obtained by periodically switching the on/off state of the laser, and applying positive or negative bias voltage. Then the current sequence can be encoded and encrypted according to the predetermined definition, and finally the encrypted image is obtained.

Figure S1a shows the optical image of the PtTe_2_/WS_2_ vdWs heterostructure device. Au electrodes are directly transferred onto PtTe_2_ and WS_2_, forming good interfacial contacts without Fermi pinning. Figure S1b shows the atomic force microscopy (AFM) images of PtTe_2_ and WS_2_, which demonstrate that the surfaces of WS_2_ and PtTe_2_ are smooth and uniform. The thicknesses of WS_2_ and PtTe_2_ are ~8.2 nm and ~55.4 nm, respectively. Raman spectra of the PtTe_2_/WS_2_ vdWs heterostructure are characterized and illustrated in Fig. S1c. In the WS_2_ region, the strong peak at 353 cm^−1^ belongs to the ${{\rm{E}}}_{{\rm{g}}1}^{2}$ phonon mode, corresponding to the in-plane atomic vibration, and the weak peak at 418 cm^−1^ is attributed to the A_1g_ mode^28^. In the PtTe_2_ region, two characteristic peaks locate near 111 cm^−1^ and 157 cm^−1^, corresponding to the *E*_*g*_ and *A*_1g_ optical phonon modes, respectively^29^. Typical vibration modes of both PtTe_2_ and WS_2_ appear in Raman spectra at the overlapping region, which indicates the successful formation of PtTe_2_/WS_2_ heterostructure.

Heterostructure with a unilateral depletion region is usually constructed by selecting a wide bandgap and a narrow or zero bandgap material, and the width of the unilateral depletion region can be adjusted by bias voltage to achieve both fast response and low dark current^30,31^. Specifically, the wide unilateral depletion region achieved by positive bias voltage increases the barrier and decreases the diffusion current. Positive bias voltage enhances the overall electric field, which can accelerate the separation of electron-hole pairs. Whereas negative bias voltage commonly leads to a narrow unilateral depletion region and increased diffusion current, resulting in an increase in dark current. Therefore, these two modes allow the device to obtain at least 4 different current levels of appropriate magnitudes. Figure 1c illustrates the distinct carrier transport behaviors under the two operating modes of PtTe_2_/WS_2_ vdWs device. Next, the mechanism therein will be further explained.

In order to study the mechanism of bias-switchable response in PtTe_2_/WS_2_ vdWs heterostructured device, the correlative energy band structure diagrams are illustrated. The electron affinity potential and band gap of WS_2_ are reported to be ~4.5 eV and 1.4 eV, respectively^32^, while the electron affinity potential of PtTe_2_ is ~5.3 eV^33^. Accordingly, Fig. S1d gives the energy band structure between PtTe_2_ and WS_2_ before contact. When forming a heterostructure, electrons transfer from WS_2_ to PtTe_2_ due to the difference in the Fermi energy level. As a consequence, the WS_2_ Fermi energy level bends upward, forming an electric field direction from WS_2_ to PtTe_2_ (Fig. 1d), and a unilateral depletion region only lies in the WS_2_ region at the equilibrium state. Figure S2 illustrates the KPFM data of the heterostructure, revealing the 82 meV contact potential difference (CPD) between the two materials upon contact. Weyl semimetal material PtTe_2_ is insensitive to the modulation of *V*_gs_ under charge shielding effect^34^, thus the gate voltage modulation mainly affects the Fermi level of WS_2_^35^.

For the case of *V*_ds_ > 0 V (Fig. 1d, left), the positive *V*_ds_ enhances the electric field, causing an overall downward shift of the energy band of WS_2_, leading to a broadening in the WS_2_ depletion region and ultimately, a complete depletion of carriers^31,36^. This makes it more difficult for the minority carriers to drift across the potential barrier, which reduces the dark current while simultaneously leading to a lower photocurrent. Photogenerated electron-hole pairs rapidly separate in the junction region under the enhanced built-in electric field. Under these circumstances, PV effect dominates the photoresponse. Therefore, low dark current and effective carrier separation jointly promote the D^*^, and the response speed of the device.

For the case of *V*_ds_ < 0 V (Fig. 1d, right), the negative *V*_ds_ makes the overall band shift of WS_2_ reduce the degree of band bending and causes a narrow depletion layer. The overall direction of the electric field is conducive to the majority carriers of WS_2_ drifting towards PtTe_2_, thus causing a large dark current. However, the holes are blocked due to a large valence band shift, and the holes gather at the heterostructure interface to produce an extra photogating effect^27^, which ultimately reduces the barrier and further results in a larger photocurrent. Therefore, PC effect plays a dominant role in this occasion. Photogenerated carriers are mainly generated in the WS_2_ region, and photogenerated electrons transit to the conduction band of PtTe_2_ by means of interlayer transition. However, the photogenerated holes are captured and accumulated by surface defects in WS_2_, while the photoelectrons in WS_2_ drift to PtTe_2_ and subsequently circulate in the external circuit under the driving external electric field. It is valuable to be noted that for positive V_ds_, the enhanced electric field extracts holes away from the surface or interface region, substantially reducing their dwell time. As a result, the defect traps have a large difficulty to capture holes. Following the principle of charge conservation within the device, these electrons quickly reach the drain and are immediately replenished from the source. In this way, the photogenerated electrons recirculate multiple times in the external circuit within their lifetime, resulting in large trap-assisted photoconductive gain and high responsivity, but the response speed is relatively slow due to the trapping prolonged carrier lifetime^37^. Therefore, the dual-mode switchable and reconfigurable device can be realized through bias-modulated energy band engineering and carrier dynamics. The device can flexibly switch the applicable response mechanism to meet the performance requirements in different application scenarios.

### Photocurrent distribution and carrier dynamics evolution under switchable photoelectric conversion modes

**Fig. 2:**
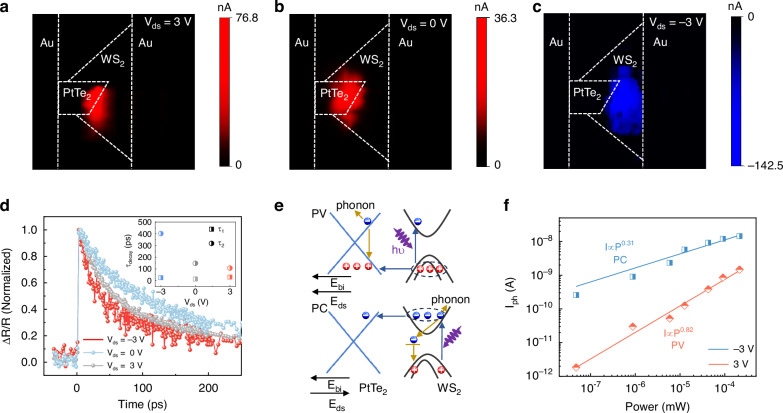
Dual mode switchable mechanism of PtTe_2_/WS_2_ heterostructure. **a**–**c** Photocurrent mappings in PtTe_2_/WS_2_ heterostructure device at *V*_ds_ = 3, 0, −3 V, respectively. **d** Ultrafast spectral response of irradiated PtTe_2_/WS_2_ heterostructure under different *V*_ds_ and the time constants of the transient absorption signal obtained by double exponential fitting. The illustration is the slow relaxation index of double exponential fitting under different *V*_ds_. **e** Schematic diagram of the carrier excitation and relaxation process of the two modes, where the blue and yellow lines represent the excitation and relaxation processes, respectively. **f** These light intensity-dependent photocurrent for dual operating modes at *V*_gs_ = −20 V

Photocurrent mapping is an effective approach to analyze the distribution of built-in electric field and photocurrent distribution in the device, and ultrafast spectra with femtosecond resolution is a universal approach to study the carrier dynamics of materials. Therefore, to further verify the above switchable response mechanism, the photocurrent mapping and the ultrafast spectra are performed, and the response speed of the device is measured, as shown in Fig. 2. Spatially resolved photocurrent mappings with the illumination of 532 nm laser at different bias voltages show distinct photocurrent distributions (Fig. 2a–c). Significant photocurrent generation in the overlapping PtTe_2_/WS_2_ region is observed at *V*_ds_ = 0 and 3 V, and the photocurrent is larger when *V*_ds_ = 3 V (Fig. 2a, b), reflecting the dominant role of the built-in electric field and PV effect. It is noted that, when the device is under reverse biased (*V*_ds_ = −3 V), the photocurrent is mainly generated at the WS_2_ region due to narrow depletion region. In this case, PC effect dominates the photoresponse in turn (Fig. 2c). These observations verify the bias-switchable photoresponse mechanism in the PtTe_2_/WS_2_ heterostructure, demonstrating the different modes of operation in Fig. 1d above.

To further validate the photoresponse mechanism and understand carrier dynamics evolution, in-situ ultrafast spectroscopy with bias voltage modulation (pump laser 410 nm, probe laser 615 nm) during device operation is conducted. Pump-probe spectra of the heterostructure region of PtTe_2_/WS_2_ are shown in Fig. 2d, and the schematic demonstration is shown in Fig. 2e. The inset within Fig. 2d shows the time constants obtained through the ultrafast spectral curve fitting at different V_ds_. The photogenerated carriers follow a biexponential decay composed of a fast and a slower lifetime component, which can be attributed to different charge transfer process and nonradiative recombination pathways, respectively. Notably, the PtTe_2_/WS_2_ heterostructure shows a significant longer lifetime at *V*_ds_ = −3 V and a shorter lifetime at *V*_ds_ = 3 V. This is in contrast to the few-layer WS_2_ region, where the lifetime of carriers increases regardless of the polarity of the applied *V*_ds_ (Fig. S3). The specific relaxation time constants and relevant fitting confidence metrics are shown in Table S1. Notably, the relative confidence intervals for all time constants are within 8%, indicating that the fitting parameters are well constrained and the extracted time constants are statistically reliable. To elucidate the potential relaxation mechanism in PtTe_2_/WS_2_ heterostructure, the energy band alignment schematic under different *V*_ds_ is given, as shown in Fig. 2e. When a positive *V*_ds_ is applied, the external electric field enhances the carrier injection efficiency, thus photogenerated holes quickly transfer to PtTe_2_ via interlayer charge transfer. This is followed by a phonon-assisted nonradiative interband recombination with a timescale of approximately ~100 ps^38^. Therefore, this process reflects that the carrier relaxation is predominantly governed by the PV effect of the heterostructure device. The situation in PtTe_2_ is similar to that of WS_2_.

In contrast, when a negative *V*_ds_ is applied, the direction of the external bias voltage opposes the built-in electric field, which suppresses the spatial separation of the carriers. As a result, the excited electrons prefer to remain near the conduction band minimum of WS_2_. At this time, shallow defect states such as sulfur vacancies in WS_2_ can trap photogenerated carriers efficiently^39^. Therefore, in this scenario, the probability of interlayer transfer is much lower than the trapping rate at these defect levels^40,41^. After this process, the trapped carriers escape from defect states and eventually recombine via delayed interband recombination, with a timescale of several hundred picoseconds, which is approximately four times longer than that in the PV mode. The long lifetime in PC mode indicates that the carrier relaxation dynamics are dominated by the defect states^42,43^. Therefore, this process exemplifies the carrier relaxation dynamics predominantly governed by the PC effect in a heterostructure device. Furthermore, the ultrafast spectra of the WS_2_ region (Fig. S2) reveal that the recovery time for both positive and negative *V*_ds_ is longer than that under zero bias, which is inconsistent with the faster relaxation dynamics in the heterojunction region. This discrepancy further highlights the critical role of the applied external bias voltage in the heterojunction in enhancing carrier separation efficiency and suppressing defect-mediated trapping.

Due to the different relaxation modes, the response of photocurrent (*I*_*ph*_) to light intensity varies under different working mechanisms. Figure 2f depicts the photocurrent (*I*_*ph*_) associated with light intensity at different V_ds_ (−3 and 3 V). *I*_*ph*_ and *P* obey a power-law relationship and are given by ${I}_{{ph}}\propto {P}^{\alpha }$, where 0 ≤ *α* ≤ 1. The index *α* provides insight into the mechanism of photocurrent generation, where a value close to zero indicates that the PC effect is dominant, while an *α* close to 1 indicates that the PV effect is dominant^44,45^. *α* values at −3 V and 3 V are 0.31 and 0.82 respectively. This also indicates that, under positive and negative V_ds_, the dominant mechanisms of PtTe_2_/WS_2_ device are PV effect and PC effect, respectively. Moreover, KPFM at different V_ds_ reveals that the CPD at *V*_ds_ = 3 V is greater than that of *V*_ds_ = −3 V, which is in line with our theory.

### Photoelectric performance and reconfigurable high-resolution imaging

Responsivity (*R*), specific detectivity (D^*^) and external quantum efficiency (*EQE*) are important parameters for evaluating the sensitivity of photodetectors. The performance of PtTe_2_/WS_2_ vdWs detector is calculated according to the following formulas:

1 $$R=({I}_{{light}}-{I}_{{dark}})/{P}_{{light}}$$

2 $${D}^{* }=R\times {{A}_{{eff}}}^{0.5}/{(2e{I}_{{dark}})}^{0.5}$$

3 $${EQE}={Rhc}/e\lambda \times 100 \%$$

**Fig. 3:**
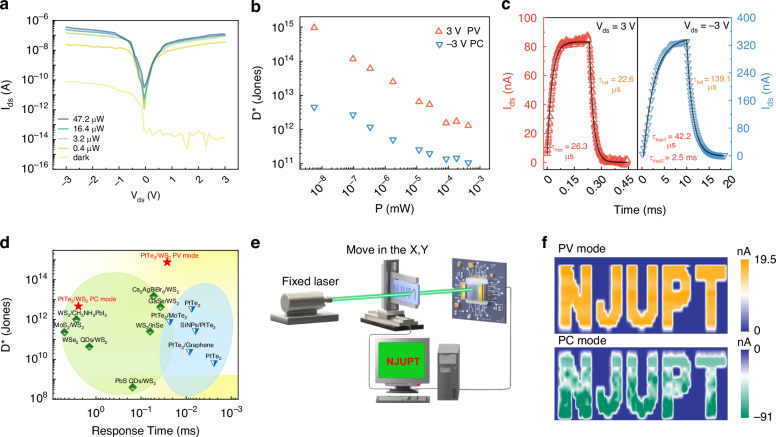
Photoelectric properties of PtTe_2_/WS_2_ heterostructure device at 532 nm. **a** Output characteristic plots of the device at *V*_gs_ = −20 V under varying light intensities. **b** The light intensity-dependent D^*^ for dual operating modes. **c** Temporal photoresponse at various *V*_ds_. **d** Comparison of the performance of PtTe_2_/WS_2_ heterostructure device with other recently reported PtTe_2_ heterostructure and WS_2_ heterostructure devices. **e** Schematic diagram of imaging system. **f** Imaging results of “NJUPT” with *V*_ds_ = −3, 3 V under 520 nm laser illumination

Where *I*_*light*_ and *I*_*dark*_ are the source-drain current under illumination and dark condition, respectively. *P*_*light*_ and *A*_*eff*_ represent the optical power and device area. To determine the optimal photoelectric testing conditions, three-dimensional maps of *R* and D^*^ under 532 nm laser illumination are acquired (Fig. S4a–d). The device exhibits the highest *R* at *V*_gs_ = 20 V, the associated large dark current leads to a significantly reduced D^*^ compared to *V*_gs_ = −20 V. Additionally, *I*-*V* curves and transfer characteristics curves under different conditions (Figs. S4e, f and S5) further corroborate this trend. Notably, the maximum photocurrent occurring at *V*_ds_ = −3 V with *V*_gs_ = −20 V (Fig. S6a) is consistent with the underlying mechanism. Therefore, *V*_gs_ = −20 V is chosen as the optimal gate voltage for subsequent dual-mode photoelectric characterizations at room temperature. Figure 3a shows the *I*-*V* curves of the PtTe_2_/WS_2_ heterostructure device in dark conditions and at different optical powers at 532 nm, ranging from 440 nW to 47.2 μW. It can be seen that the dark current can be reduced by 4 orders of magnitude when the *V*_ds_ has been switched from −3 V to 3 V due to enhanced depletion region and built-in electric field, thereby contributing outstanding D^*^ when operated under positive *V*_ds_.

Figure 3b shows the D^*^ extracted at 532 nm under different incident light intensities. The device exhibits a maximum D^*^ of 9.42 × 10^14^ Jones under the incident optical power of ~5.7 pW and V_ds_ of 3 V. While the maximum D^*^ at −3 V at the same optical power is only 4.68 × 10^12^ Jones. When *V*_ds_ = 3 V, the maximum *R* and *EQE* of the PtTe_2_/WS_2_ vdWs device are 0.60 A W^−1^ and 139.51%, respectively. The maximum *R* and *EQE* at −3 V are 1.37 A W^−1^ and 263.67%, respectively (Fig. S6b,c). In addition, as shown in Fig. S6d, the device shows a high on/off ratio of up to 10^5^ at *V*_ds_ = 3 V. When the power of the incident laser is ~5.7 pW, the device can still maintain an on/off ratio of >10^2^.

Figure 3c depicts the response speeds at different *V*_ds_. The rise and fall times in PV mode (*V*_ds_ = 3 V, *V*_gs_ = −20 V) are 26.3 μs and 22.6 μs, respectively. The rise process of photoresponse in PC mode (*V*_ds_ = −3 V, *V*_gs_ = −20 V) contains two processes (τ_rise1_ ~ 42.2 μs, τ_rise2_ ~ 2.45 ms). The fast process corresponds to free carrier generation, and the slower process is of carrier trapping in shallow defect states, respectively^46,47^. And the fall time in PC mode is 139.1 μs (τ_fall_), which is much slower than that in PV mode. The difference in response speed between the above two modes is mainly due to the adverse defect trapping and the reduced built-in electric field^48^. Therefore, combining the millisecond-level response in the PC mode and the strong sublinear power dependence, we believe that the above phenomenon is mainly the consequence of defect-mediated carrier dynamics. And in this case, the main working principle of the device is the PC effect. Moreover, the faster response at *V*_ds_ > 0 V reflects more efficient carrier extraction and less capture. Compared to typical 2D vdWs heterostructure devices (Fig. 3d and Table S2) and other mode-switchable devices (Table S3) reported previously, the device under PV mode exhibits excellent sensitivity and high-speed response. The D^*^ of our PtTe_2_/WS_2_ device is more than two orders of magnitude higher than other vdWs heterostructure devices. This enhancement can be attributed to the very low dark current (1.27 × 10^−14^A), due to the existence of a wide depletion region and strong built-in electric field. In addition, time-dependent photoresponse in Fig. S6e shows that the photocurrent response increases with the optical power from 10 nW to 308 μW since the number of photogenerated carriers is proportional to the incident photon flux^49^. The spectral response of the PtTe_2_/WS_2_ device is also measured. As shown in Fig. S6f, it can be seen that the device can respond from 420 to 1000 nm, which indicates that the device has a great potential for broadband detection. Moreover, the performance of PtTe_2_/WS_2_ heterostructure device is indeed substantially improved compared to the bare WS_2_ device (Fig. S7). The photoelectric performance of bare PtTe_2_ is shown in Fig. S8, and the influence on device’s photoelectric performance by changing the thicknesses of WS_2_ and PtTe_2_ is discussed respectively (Fig. S9).

The exceptional photoresponse of PtTe_2_/WS_2_ heterostructure device, coupled with high *R* and D^*^, highlights its potential for imaging applications. Figure 3e illustrates the schematic of the single-point imaging system, where a mask plate with a hollow imaging pattern is placed in the path of the laser light path, and the mask plate is subjected to regular *X*-*Y* axis motion in the 2D plane. Laser beam passes through these patterns and illuminates the PtTe_2_/WS_2_ device, resulting in considerable photocurrents. By spatially resolving photocurrent mapping, we generate a 2D contrast image that reproduces the hollow patterns on the mask. Figure 3f shows the imaging results achieved at 532 nm with different *V*_ds_ (−3 V and 3 V), with utilizing the patterns with the letters “NJUPT” and distinguishing them from the background with clearly distinguishable boundaries. These high-resolution images contain 420 × 80 pixels, highlighting the reliable imaging capability of the PtTe_2_/WS_2_ device. It is worth noted that the imaging quality of *V*_ds_ = 3 V is higher than that of *V*_ds_ = −3 V because the former is dominated by the PV mode with higher D^*^ and SNR. The device can flexibly switch imaging modes according to different application scenarios. PV mode is suitable for conventional high-resolution imaging, while PC mode is good for weak light imaging if large photogain and large photocurrent are required. Figure S10 is a device stability test, showing that the device can maintain a stable photocurrent without any degradation after 300 s test, which indicates the excellent stability of PtTe_2_/WS_2_ device.

Moreover, the power consumption formula is described as:

4 $${E}_{{on}}=U\times I\times \Delta t$$

where *I* is the peak value of generated impulse voltage, Δ*t* is the spike pulse width, and *U* is the applied drain-source voltage. We calculated the power consumption for each event under illuminated and non-illuminated conditions was 1 and 2 fJ, respectively, which is comparable to the performance of the best-in-class, high-speed 2D photodetectors (Fig. S11).

### Image encryption demonstration of proof-of-concept application

Optical logic gates, such as XNOR and NOR gates, leveraging nonlinear optical effects and enabling real-time data processing, are important components for optical encryption systems. For information decryption, the correct keys must be supplied to configure the logic gates properly to retrieve the original information. This highlights the importance of logic gate design in securing optical communication. Based on the previous discussion of the device’s mechanism and phenomena, this PtTe_2_/WS_2_ heterostructure device can reproduce the functions of logical gates such as XNOR, XOR, and NOR.

**Fig. 4:**
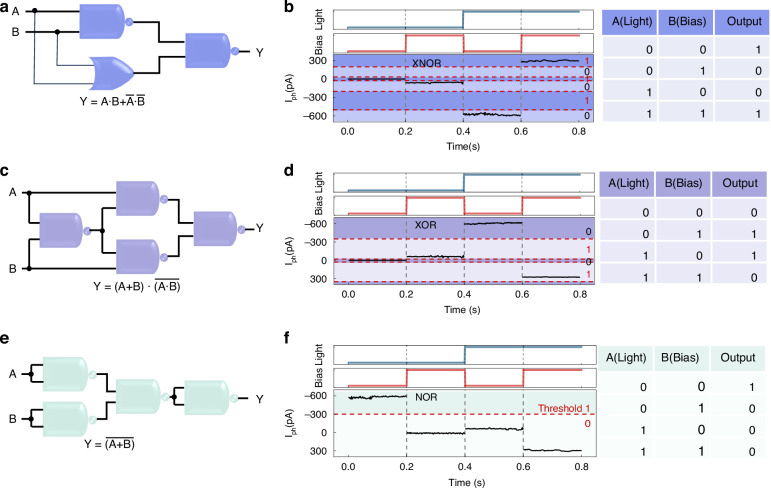
Demonstration of the principle of logic gates simulated by PtTe_2_/WS_2_ photo-transistors. **a**, **c**, **e** The equivalent circuit diagrams of the XNOR, XOR, and NOR logic gates, respectively. **b**, **d**, **f** The channel A,B inputs and corresponding output optical signals of XNOR, XOR and NOR, respectively. XNOR sets the absolute value of the photocurrent to be less than 2 × 10^−11^A or within the range of 2.5 × 10^−10^A to 5 × 10^−10^A as “1”, the light irradiation as “1”, and the negative *V*_ds_ as “1”. XOR sets the absolute value of the photocurrent greater than 3.5 × 10^−10^A or less than 2 × 10^−11^A as “0”, light irradiation as “1”, and positive *V*_ds_ as “1”. NOR sets the photocurrent less than −3 × 10^−10^A as “1”, no light as “1”, and negative *V*_ds_ as “1”

The logic truth tables in Fig. 4 are derived from experimentally measured drain-current responses under different input combinations, with logic levels assigned using predetermined current thresholds. The actual fastest mode switching time of the devices is 191 μs (as shown in Fig. S12). For the XNOR logic gate, as shown in Fig. 4a, its equivalent circuit is implemented by combining NOT, AND, and OR basic logic gates to realize the logic expression $\mathrm{Y}=\mathrm{A}\cdot \mathrm{B}+\bar{\mathrm{A}}\cdot \bar{\mathrm{B}}$. The input signal A is used to control the optical source, representing the binary states “0” or “1,” while the signal B modulates the polarity of the bias voltage to encode logical “0” or “1”, as shown in Fig. 4b. These two input signals form four possible combinations. The encryption application discussed in this article can be implemented within the range of 0.1 μW to 7 μW of the actual received light intensity by the device. This is done to ensure that the dark current in the PV mode is less than that in the PC mode. The phototransistor’s output photocurrent (*I*_*ph*_) varies in amplitude depending on the input states, and a predefined current threshold (2.5 × 10^−10^A < |*I*_*ph*_| < 5 × 10^−10^A or |*I*_*ph*_| < 2 × 10^−11^A) is used to map the output current to binary logic: when the photocurrent exceeds this threshold, the output is interpreted as logical “1”; otherwise, it is interpreted as “0”. Specifically, under input conditions (0, 0) and (1, 1), the output photocurrent is relatively high and classified as logical “1,” whereas for inputs (0, 1) and (1, 0), the current is lower and interpreted as logical “0”. This results in an output sequence of “1001”, thereby realizing the function of the XNOR logic gate. The implementation methods of the logic gates XOR (Fig. 4c, d) and NOR (Fig. 4e, f) are similar, by defining the binary numbers corresponding to different levels of current. The current thresholds defining logic “0/1” are chosen to maximize the noise/operational margin while maintaining sufficiently wide acceptance regions for robust logic-state discrimination. For example, for XOR, logic “0” is identified as *I*_ph_ > 3.5 × 10^−10^A or *I*_ph_ < 2 × 10^−11^ A; the measured “0” output levels (∼5.9 × 10^−10^ A and ∼1 × 10^−13^ A) are well separated from the thresholds, minimizing misclassification. Moreover, this device can also simulate the operation of sequential circuits to interpolate a sequence and thereby achieve another form of encryption.

**Fig. 5:**
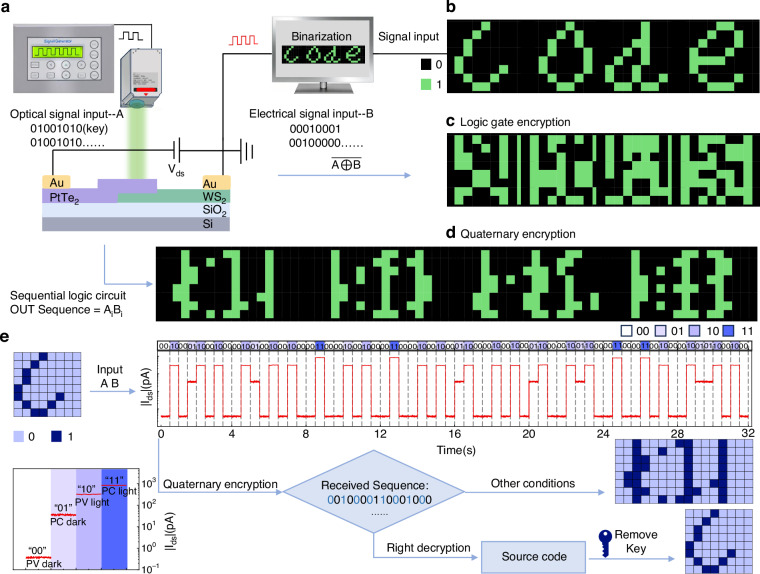
Encrypted image of dual-mode switchable vdWs device. **a** Schematic of two encrypted signals input heterostructure device (“*i*” is the position of the sequence element (*i* = 1~*n*) and “*n*” is the length of the sequence.). **b** Original image. **c** Image after encryption by logic gate. **d** Image after four-state currents encryption. **e** These specific quaternary encryption and decryption process of the lowercase letter “c” (blue numbers belong to A; black numbers belong to B; Key sequence is A.)

Building upon the dual-mode response mechanism and the photoelectric logic gate functionalities, an optical communication encryption system is constructed, driven by a cyclic sequence defined by an 8-bit pre-configurable key. In this system, the four photocurrent states are assigned binary encodings to implement XNOR, NOR, XOR logic gates and four-state current encryption, with the working principle shown in Fig. 5a.

In this device, the light intensity and bias voltage serve as two independent information channels, denoted as Channel A and Channel B, respectively. Specifically, two sets of binary source signals are employed to control the light source (on/off) and the bias voltage polarity (positive/negative) via separate controllers. Logical definitions are assigned as follows: light on is “1” for A sequence, positive bias voltage is “1” for B sequence, while negative bias voltage and no light are defined as “0”. In Channel A, a cyclic sequence of an 8-bit binary key is used as the light intensity control signal. In Channel B, each row of the original image in Fig. 5b is scanned, and pixels containing graphical elements are marked as “1” to form the bias voltage control sequence. The combination of light intensity and bias voltage drives the PtTe_2_/WS_2_ phototransistor to generate a photocurrent sequence, which serves as the encrypted information.

For the logic gate-based encryption mode (Fig. 5c), the XNOR operation is taken as an example. According to the logic rules defined in Fig. 4b, a bitwise XNOR is performed between the binary signals from Channel A and Channel B, and the resulting binary code sequence constitutes the encrypted key stream. In addition to the encryption of letter images, we have also implemented the encryption of other graphics, as shown in Fig. S12a. The decryption process under the XNOR mode, shown in Fig. S12b, involves decoding the photocurrent states at the receiver end based on the XNOR rule (Fig. S12c) to reconstruct the binary code sequence and the encrypted image. The original data can be recovered by determining the graphical point positions and executing an XNOR operation with the known key.

In the four-state current encryption mode (Fig. 5d), Channel A serves as the interpolation sequence, and each bit from Channel B is inserted after the corresponding bit in Channel A, forming the final encryption sequence. Every two consecutive bits define a current state, which can be implemented via sequential logic circuits. The encrypted image is then generated by remapping rows with graphical points marked as “1”.

It is noted that the original sequence can be rearranged into an image according to the rule that every line with a graphic point is considered as “1”. For the output signal with four-state current encryption, the specific transmission and receiving process of the lowercase letter “c” is shown in Fig. 5e, while the specific decryption process is shown in Fig. S12c. First, the lowercase letter “c” is encoded into the original sequence and then input to the device through the laser sequence containing the key information, and then the absolute value operation is performed on the generated photocurrent sequence. Next, according to Fig. 1c, the corresponding situations of “00”, “01”, “10” and “11” are encoded to obtain the password sequence, and the encrypted image is further obtained. After receiving the encrypted image, the receiver restores the encryption sequence and then removes the key sequence from the code sequence according to the order of interpolation. The original sequence can be obtained, and the original image can be reproduced. Pixel correlation coefficient is one of the important indicators to measure the quality of encrypted images, and its function is to quantify the linear correlation of adjacent pixels^50^. In addition, the vertical, horizontal and diagonal components of the adjacent pixel correlation coefficient of this encryption technology are 0.0702, 0.0201, and −0.0038, respectively, all of which are close to 0 (The pixel correlations of the original matrix are 0.9388, 0.9044, 0.8943, respectively). The vertical, horizontal and diagonal components of XNOR encryption are −0.0947, −0.0612, and −0.0380, respectively, and its average correlation coefficient of 0.0646 is twice as large as four-state encryption (0.0288). This indicates the lower correlation between the original image and the encrypted image of four-state encryption, and its distinguished image encryption capability. These demonstrations establish a device-level physical-layer obfuscation primitive enabled by multi-state responses, intended to complement standard cryptographic protocols. Notably, the encoded output states are highly reproducible (coefficient of variation (CV) ≤ 7%) and remain stable over 6 months (variation ≤ 4%) (Fig. S14), and conservative thresholds with sufficient margins are used to ensure robust decoding. Moreover, by applying the relevant knowledge of probability and statistics, we justify that the data obtained from repeated on/off experiments are used to calculate the CV corresponding to the 95% confidence interval, and the hypothetical principle is used to prove that the chosen operating point simultaneously minimizes the energy consumed during each readout and maintains a high detection probability at a low false alarm rate (Fig. S15).

## Discussion

In summary, we report a dual-mode switchable high-performance PtTe_2_/WS_2_ phototransistor, demonstrating four stable photocurrent level states that enable the construction of advanced phototransistors for secure optical communication. There are two operating mechanisms within a single phototransistor: the PV effect under positive *V*_ds_ and the PC effect under negative *V*_ds_. In PC mode, the significant weakening of the energy band bending and depletion region at the heterogeneous interface promotes the photoconductivity gain. In PV mode, the E_bi_ and energy band bending enhancement produce a high D^*^ of 9.42 × 10^14^ Jones with a fast response. The photocurrents generated by these two independent operating mechanisms are distinguishable by orders of magnitude in photocurrent and dark current values. Therefore, the four output states are determined by the combination of incident light and *V*_ds_, which allows the decryption of the information by means of a single phototransistor. This development can be applied to secure optical communications based on optical response mode switching. It is expected that the color image encryption can be realized in the future by binarizing the image and solving the problem of the expansion of the corresponding RGB sequence after the four-state encryption expands the gray scale sequence. Moreover, we briefly described the potential array fabrication and integration process (Figs. S16 and S17). In addition, the device is expected to be used for information encryption in the near-infrared communication band or mid-wave infrared by virtue of the broadband absorption of 2D semi-metallic materials.

## Materials and methods

### Preparation of PtTe_2_/WS_2_ vdW heterostructure

PtTe_2_ and WS_2_ bulk crystals (provided by Taizhou SUNANO New Energy Co., Ltd) are stripped of flake seeds and placed on Nitto-blue film tape, and then multilayer PtTe_2_ and a few-layer WS_2_ are prepared on Polydimethylsiloxane (PDMS) by the mechanical stripping method. WS_2_ is first pressed onto Si (285 nm thick SiO_2_) substrate at room temperature. Then waiting 2–3 min for WS_2_ to fit with the substrate, and then lifting the PDMS to complete the transfer of WS_2_. Next, PtTe_2_ is transferred at 80 °C, and the PtTe_2_ is selectively aligned with WS_2_ through a 3D positioning system-assisted optical microscope (Olympus-BX51), and then the PtTe_2_/WS_2_ heterostructure is completed by lifting the PDMS at 80 °C until PtTe_2_ and WS_2_ were bonded.

### Device fabrication

Au electrode arrays are prepared on a pure Si substrate by mask. The specific production process is as follows: First, the pure Si substrate is cut to the appropriate size. Use a sterilized toothpick to apply the high-temperature-resistant glue on the purchased 200-wire copper net, and adjust the mesh as needed. Then, the Si substrate with copper mesh is fixed on the electron beam evaporation (EBE) sample storage platform with heat-resistant double-sided tape, and EBE sputtering Au is performed. Au electrodes of appropriate length are cut using a probe base and a steel needle with a tip diameter of 1 µm. Using a steel needle with a tip diameter of 15 µm and attach an indium gallium alloy to the needle tip to bond the electrode and transfer it to both sides of the target layer. Laying the sides are flat, leaving the channel of a certain width. In this process, the heterostructure is not affected by other operations. The electrodes on both sides of the device are used as source and drain, and the bottom Si p++ is used as gate to complete the preparation of the device.

### Characterization of PtTe_2_/WS_2_ heterostructure

AFM (conventional and high resolution) is performed by a Bruker Dimensions Icon under ambient conditions. Raman spectroscopy was performed using a combination of a Princeton Instrument SP-2560 spectrometer and a silicon CCD camera (Princeton Instrument PYL-1300BXD) with excitation provided by a 488 nm laser. In the ultrafast Spectroscopy, using the transient absorption spectroscopy system, a 410 nm/615 nm focused laser beam is used to pump/probe the corresponding region of the device.

### Optoelectrical measurements

The electrical performance of the phototransistor is tested using the semiconductor device analyzer Fs-Pro at room temperature. The photoelectric detection experiment uses a laser with an operating wavelength of 532 nm. The incident laser power is adjusted by an attenuating plate. All electrical properties are measured at room temperature. The photocurrent mapping experiment is carried out using the advanced photocurrent imaging equipment, Mstarter 200 high-precision photocurrent scanning test microscope, which can realize single pixel imaging and scanning photocurrent mapping measurement, where the laser wavelength is 520 nm.

## Supplementary information

Supporting information for “Dual-mode Switchable and Reconfigurable Van der Waals Phototransistor for Multi-state Image Encryption”

## Data Availability

The data that support the findings of this study are available from the corresponding author upon reasonable request.
