# Supplementary material for: Dual-mode switchable and reconfigurable Van der Waals phototransistor for multi-state image encryption
Source: Light Sci Appl. 2026 Jul 1;15:299. doi: 10.1038/s41377-026-02358-7 (PMC13324172; doi:10.1038/s41377-026-02358-7)
Supplement: Supplementary file 1 — Supporting information for “Dual-mode Switchable and Reconfigurable Van der Waals Phototransistor for Multi-state Image Encryption” [file 41377_2026_2358_MOESM1_ESM.docx]

**Supporting information for “Dual-mode Switchable and Reconfigurable Van der Waals Phototransistor for Multi-state Image Encryption”**

Yuanfang Yu^1^, Senyao Tang^1^, Nanjie Jiang^1^, Yuwei Zhang^2^, Xiaorui Jin^1^, Jiaxin Gong^3^, Huijuan Zhao^1^, Anran Wang^3^, Dongyang Wan^2^, Zhenhua Ni^2^, Xinran Wang^4^, Li Gao*^1,3^

^1^State Key Laboratory of Flexible Electronics (LoFE) & Institute of Advanced Materials (IAM), School of Materials Science and Engineering, Nanjing University of Posts and Telecommunications, Nanjing 210023, China.

^2^School of Physics and Key Laboratory of Quantum Materials and Devices of Ministry of Education, Southeast University, Nanjing 211189, China.

^3^School of Science, Jiangsu Key Laboratory of Quantum Computing Science and Devices, Nanjing University of Posts and Telecommunications, Nanjing 210023, China.

^4^School of Integrated Circuits, Nanjing University, Suzhou 215163, China.

**Author to whom correspondence should be addressed:** iamlgao@njupt.edu.cn





**Figure S1.** Correlation characterization of PtTe_2_/WS_2_ heterostructure device. **a** Optical microscopy photo of the device. **b** AFM image of the device, the extension line is the thickness of PtTe_2_ and WS_2_ corresponding to the delineated region, the blue line is the WS_2_ region, and the orange line is the PtTe_2_ region. **c** The Raman graph of the device. **d** Energy band arrangement of the PtTe_2_/WS_2_ heterostructure before contact and schematic diagram of PtTe_2_/WS_2_ heterostructure device and energy band arrangement of the PtTe_2_/WS_2_ heterostructure after contact.


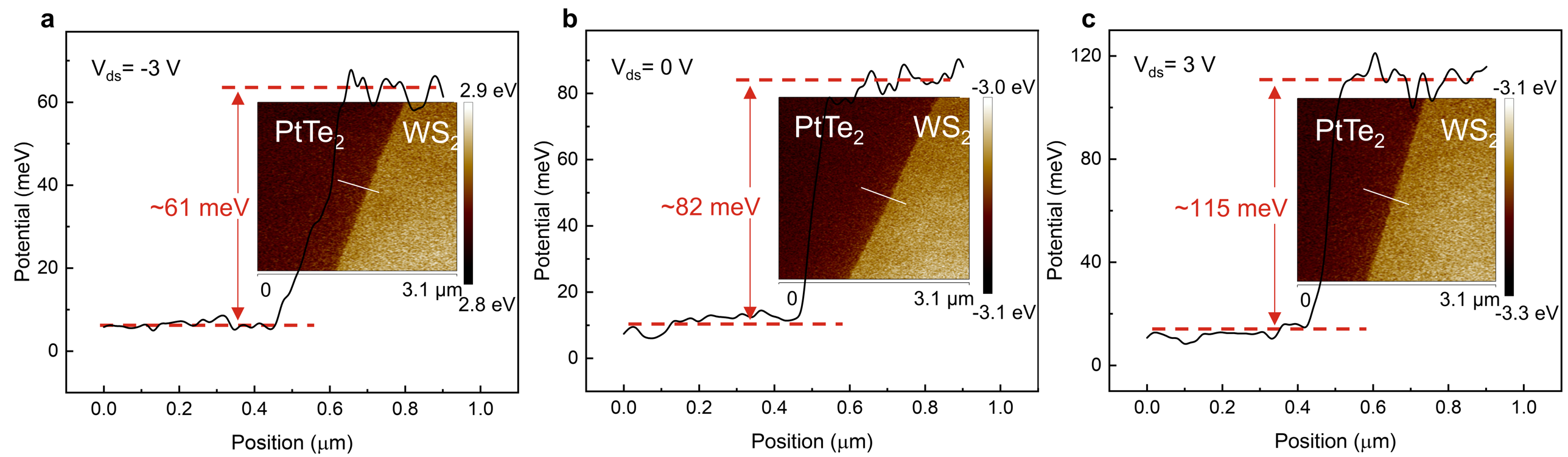


**Figure S2.** The KPFM of PtTe_2_/WS_2_ device at different V_ds_.


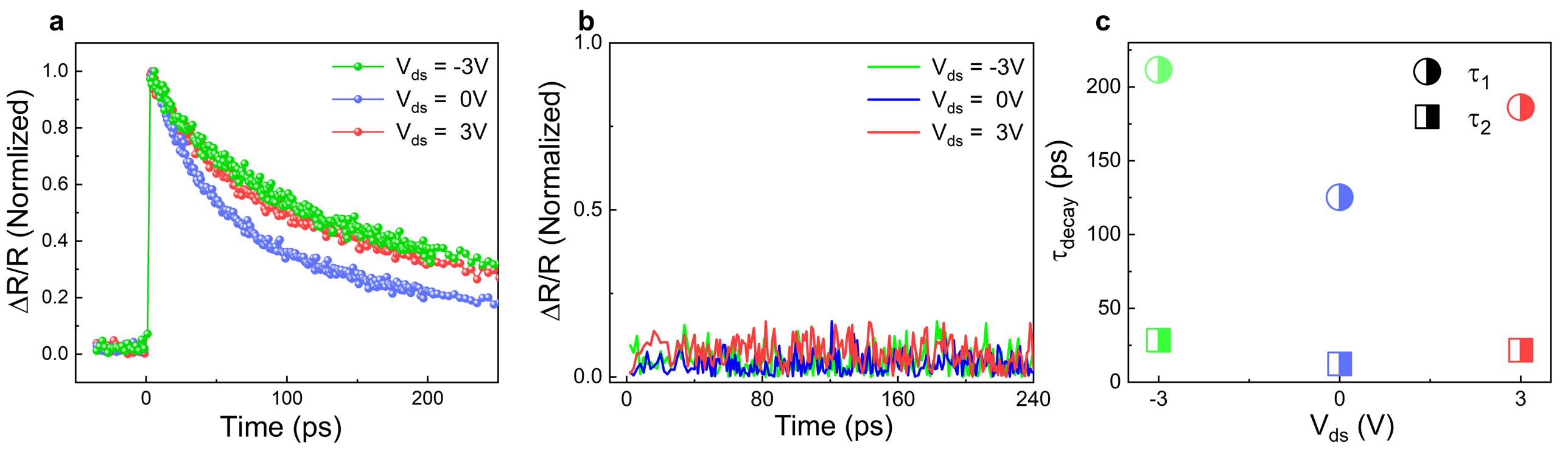


**Figure S3.** Ultrafast spectra of separately irradiated **a** WS_2_ region and **b** PtTe_2_ region at different V_ds_. **c** Relaxation time constants of ultrafast spectrum.





**Figure S4.** Three-dimensional intensity plots of **a** ,**b** R and **c** ,**d** D^*^ for V_ds_ = -3 V, 3 V and different gate voltages at varying light intensities. **e** ,**f** Transfer curves for V_ds_ = -3 V, 3 V at different light intensities.


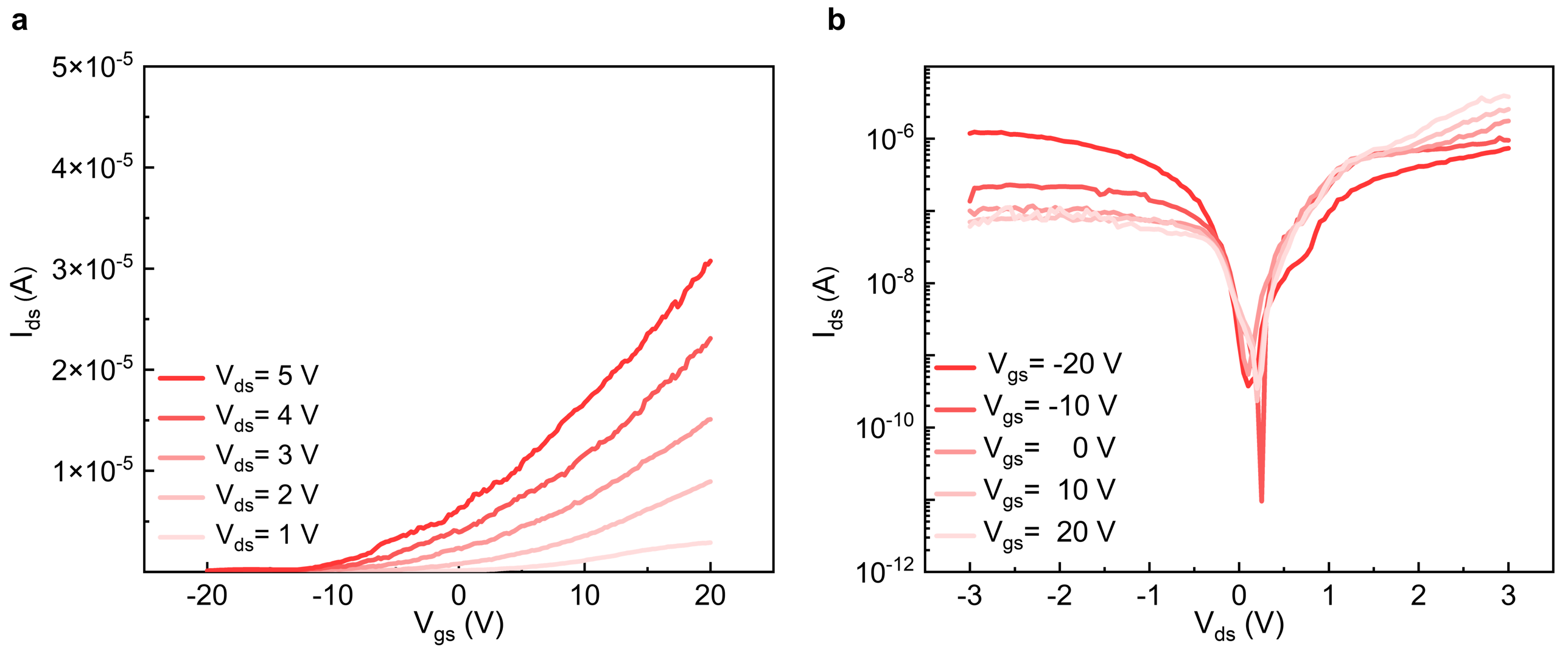


**Figure S5.** **a** Transfer curves of PtTe_2_/WS_2_ heterostructure at different V_ds_. **b** Output characteristic curves at different V_gs_ (with 532nm laser of 6.86 μW).





**Figure S6. a** PtTe_2_/WS_2_ heterostructure mapping images of output characteristic plots at V_gs_ = -20 V as functions of light intensity. The light intensity-dependent **b** responsivity and **c** external quantum efficiency at V_gs_ = -20 V for the two operating modes, respectively. **d** The light intensity-dependent on/off ratio at V_gs_ = -20 V for the two operating modes. **e** Time-resolved photoresponse measured at 532 nm laser at different optical power densities at V_ds_ = 3 V and V_gs_ = -20 V. **f** PtTe_2_/WS_2_ heterostructure photocurrent at 420-1000 nm at different wavelengths with optical power of 100 μW, V_ds_ = 3 V and V_gs_ = -20 V.





**Figure S7.** **a** Output characteristic plots of fewer layer WS_2_ device. **b-d** are *R*, *D^*^*, *EQE* of fewer layer WS_2_ devices at V_ds_= 3 V and V_gs_= -20 V as a function of optical power, respectively.


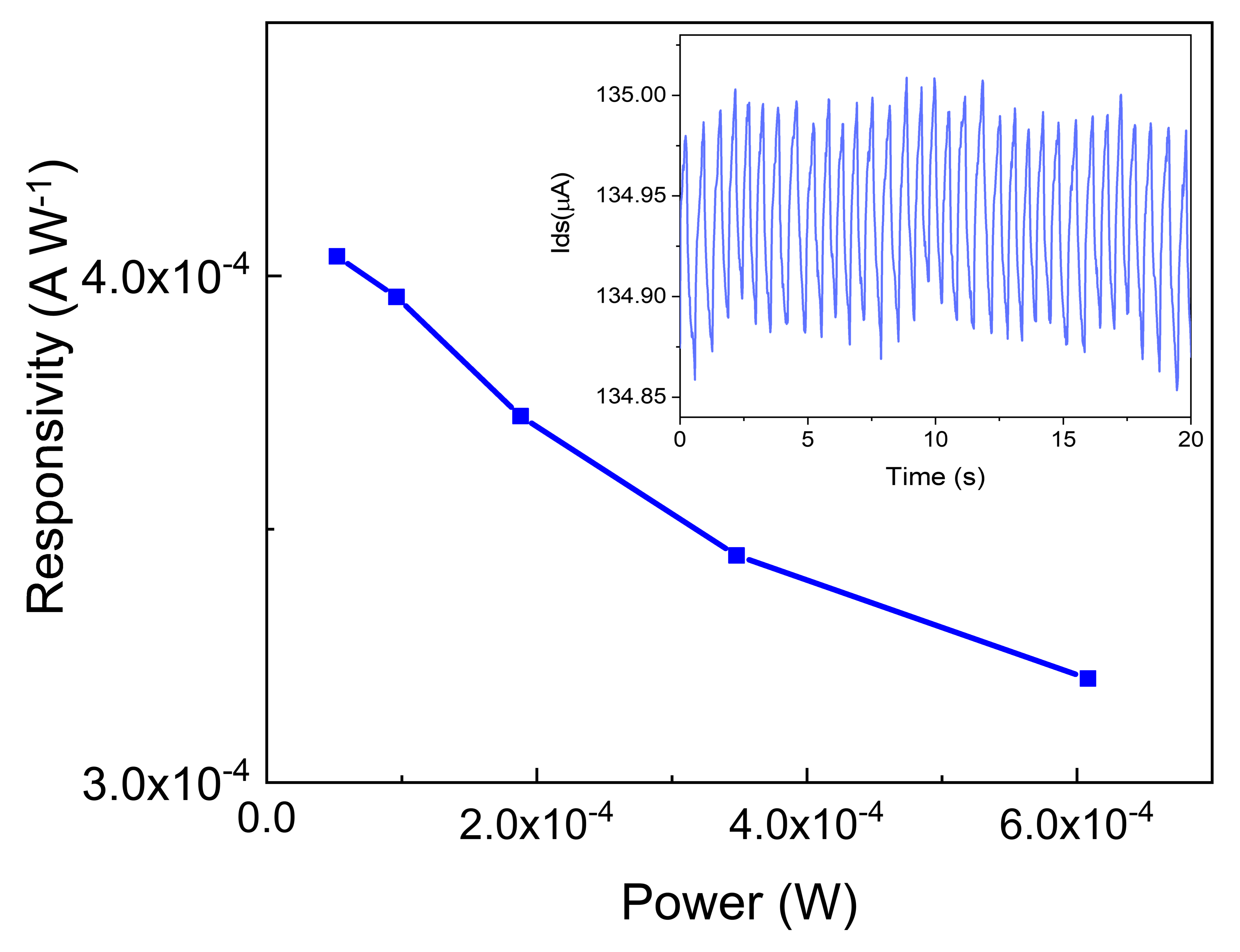


**Figure S8.** The photoelectric performance of the bare PtTe_2_ device at 532 nm.


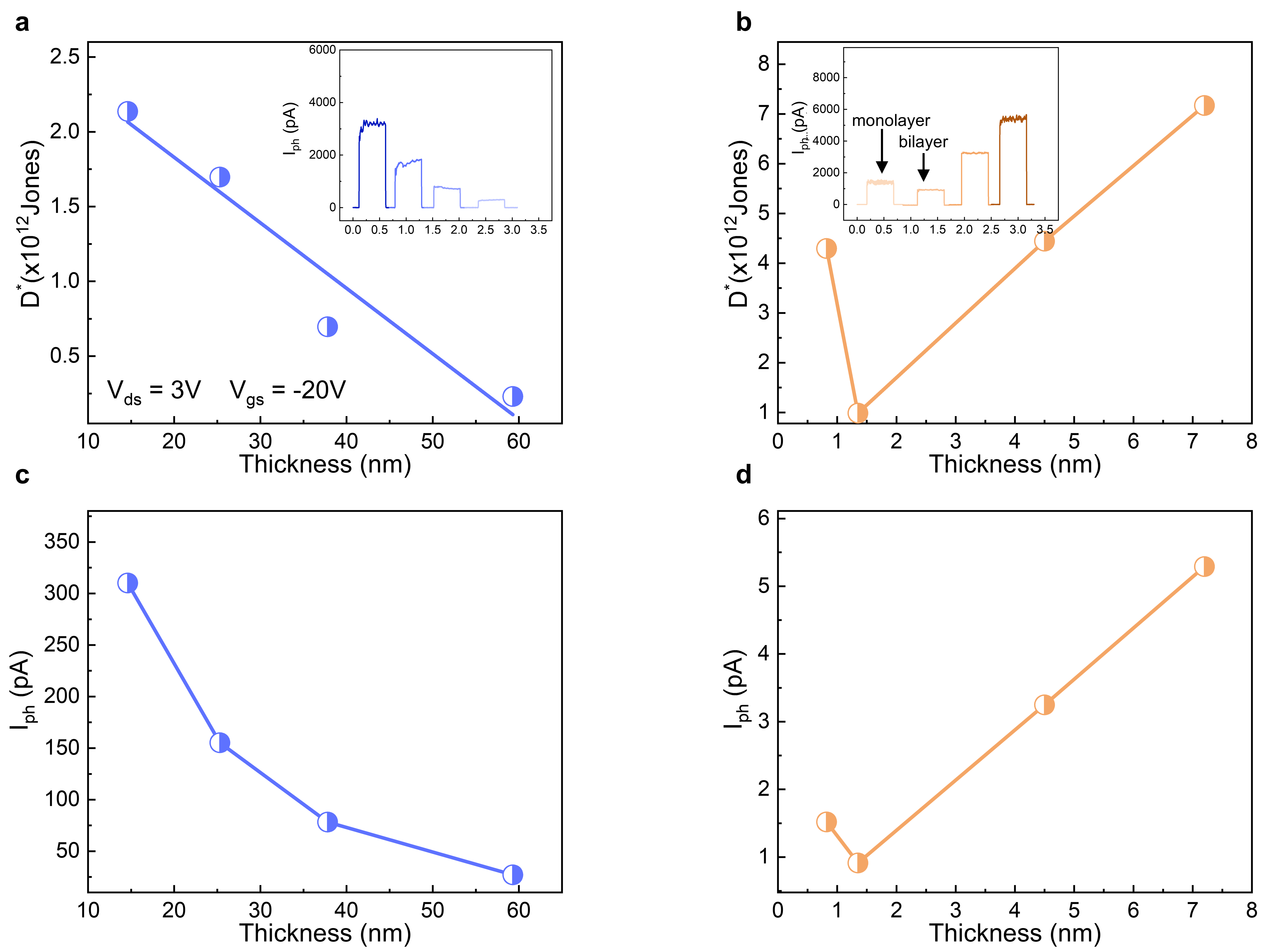


**Figure S9.** D^*^ of the PtTe_2_/WS_2_ device with varying the thicknesses of PtTe_2_ a) and WS_2_ b), respectively. Photocurrent of the PtTe_2_/WS_2_ device with varying the thicknesses of PtTe_2_ c) and WS_2_ d), respectively.

The photoresponse of the PtTe_2_/WS_2_ device is thickness-dependent for both constituents. Increasing the PtTe₂ thickness reduces the photocurrent because the optical field reaching the junction is attenuated by the thicker semimetal layer (shorter optical penetration, stronger screening), and the built-in field in the vertical junction is more effectively screened, shrinking the effective depletion region in the underlying semiconductor; both effects suppress carrier generation/separation at the active interface.

Monolayer WS_2_ possesses a direct bandgap with strong excitonic absorption, whereas few-/multilayer WS_2_ evolves toward an indirect bandgap. From a detector standpoint, however, two opposing trends compete: For monolayer, the larger photocurrent than bilayer primarily because its bandgap is direct: interband absorption proceeds via vertical, momentum-conserving transitions that do not require phonon assistance, which increases the photogeneration yield per absorbed photon. By contrast, bilayer/few-layer becomes indirect bandgap, so light absorption becomes the dominate role in generate the photocurrent. As the thickness is increased, the photocurrent increases monotonically.


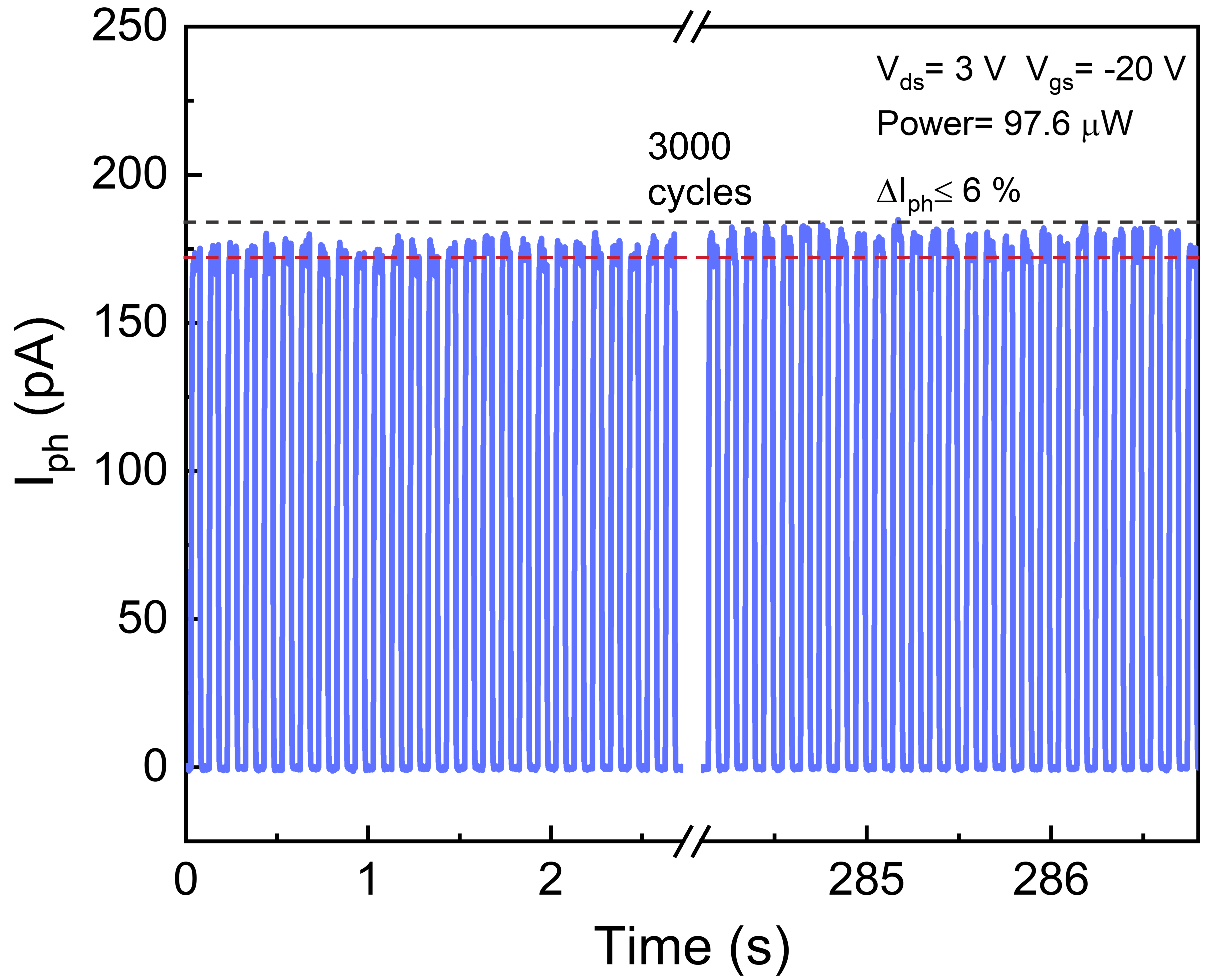


**Figure S10.** The stability test of 3000 cyces over 300 s under illumination power of 97.6 μW by a 532 nm laser at V_ds_ = 3 V and V_gs_ = -20 V. The current oscillated between the maximum value (182.02 pA) and minimum value (171.44 pA) within a variation of 6 %, which is on the same scale as the power tolerance of the fast-modulated laser of ~5 %.


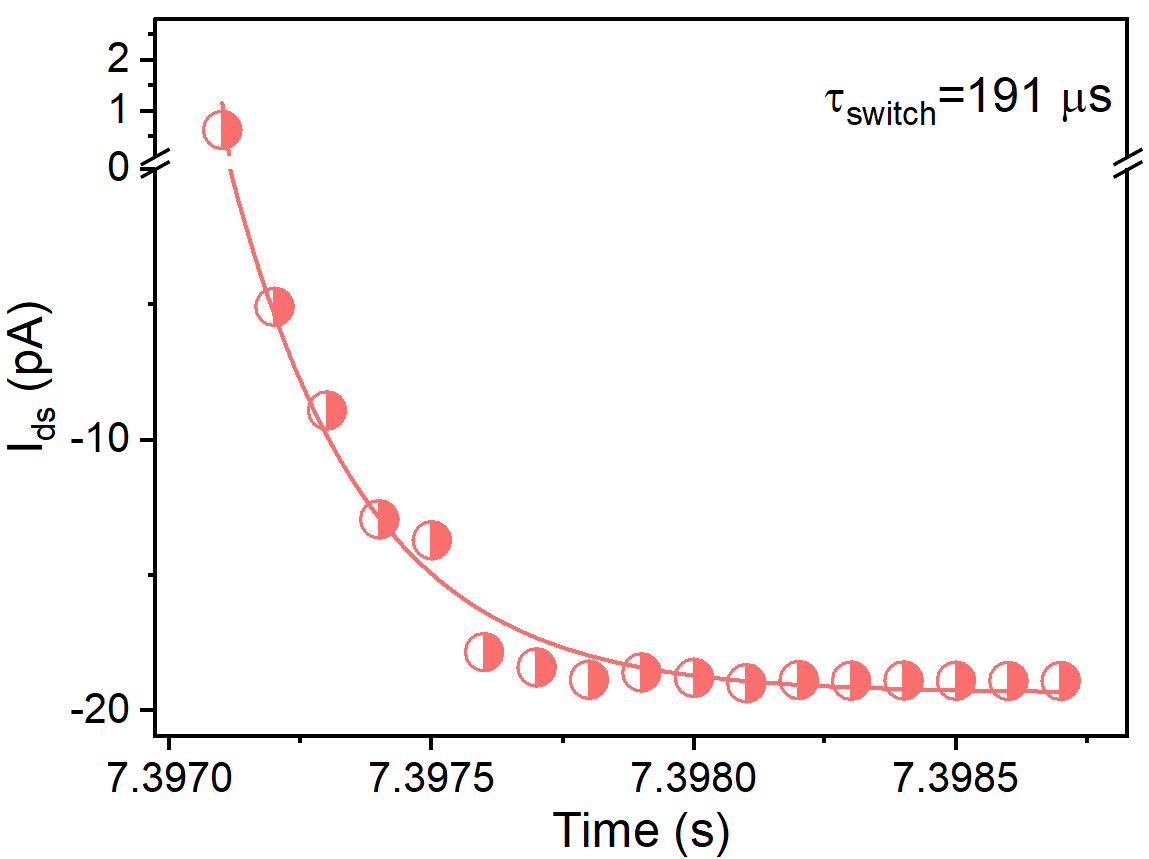


**Figure S11.** The switch speed for mode switching (PC/PV).


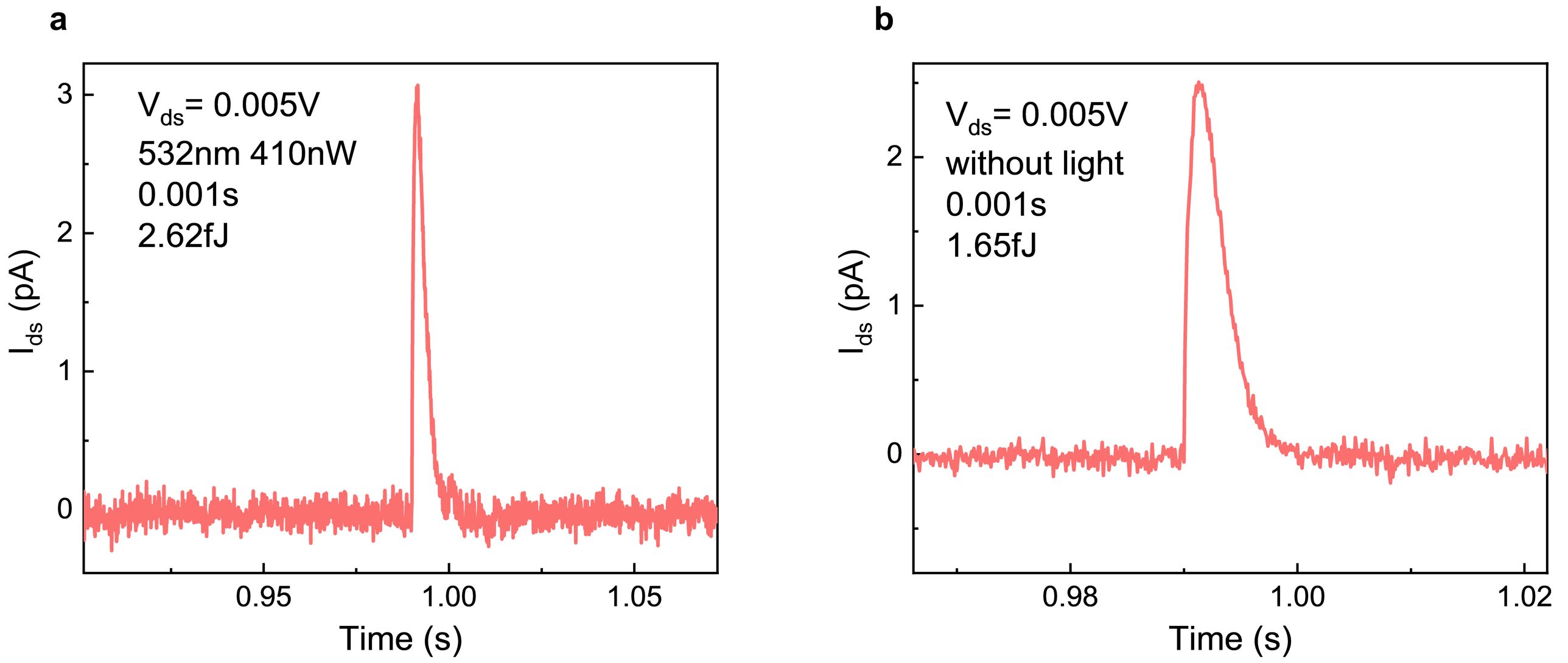


**Figure S12.** Pulse triggering current (a) under light illumination and under dark condition (b).

In the experiment, when the applied voltage was decreased to 0.5 mV with a spike pulse of 1 ms, the obvious impulse voltage still can be seen with a value of 2.61 × 10^-12^ A, which exhibits power consumption per event of 1.65 fJ.

Since the applied positive bias is in the same direction as the built-in electric field, adding light is equivalent to adding an extra voltage, so the energy consumption without adding light is higher. It already falls into a sub-picojoule regime that is considered low for logic/encryption devices or photodetectors.^1-3^ These set an aspirational floor but target different operating modalities than our bias-reconfigurable dual-mode detector. Thus, our 1.65 fJ/event is meaningfully low for encryption-oriented logic.





**Figure S13.** **a** Logic gate encryption and four-state current encryption for torch graphics. **b** XNOR decryption process. **c** Four-state current decryption process.


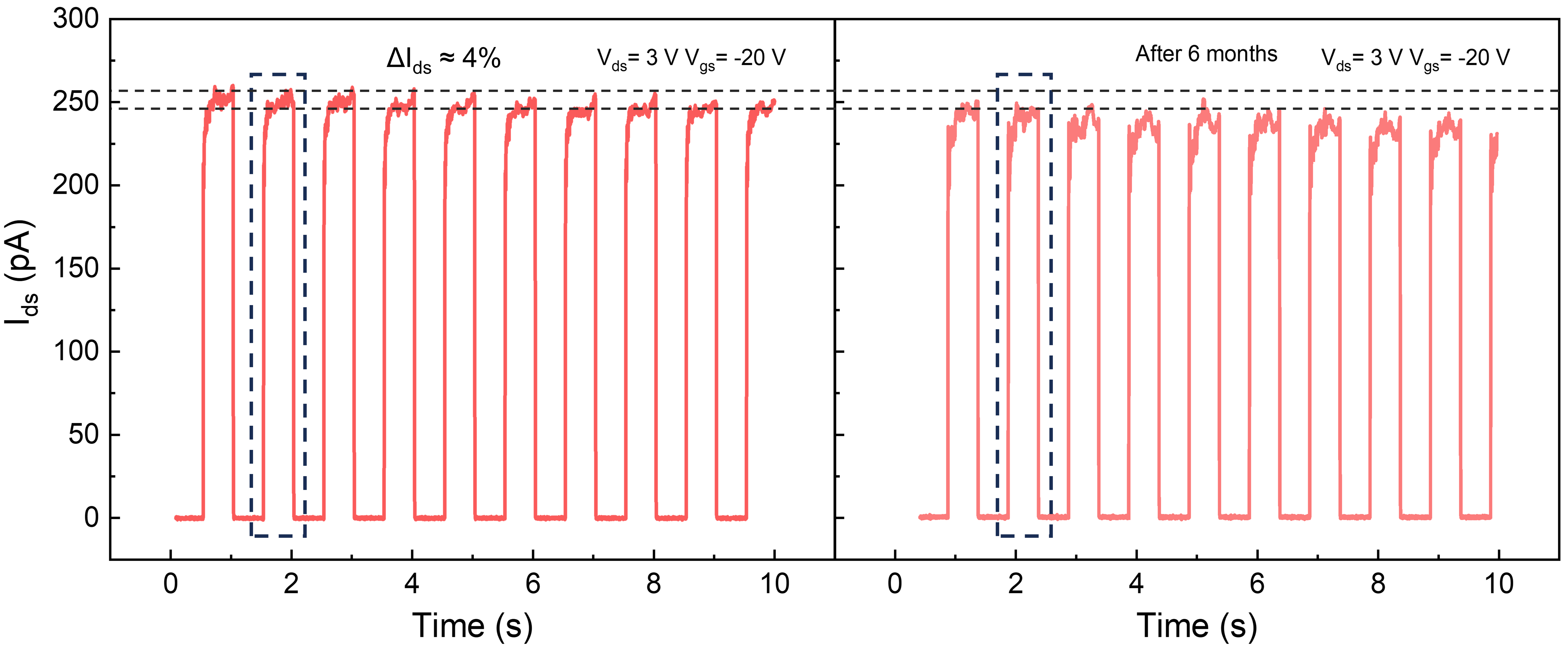


**Figure S14.** Time-resolved photoresponse of the device and the time-domain response tested after 6 months. The average photocurrent of the former is around 248.8 pA, while that of the latter is 240.7 pA.


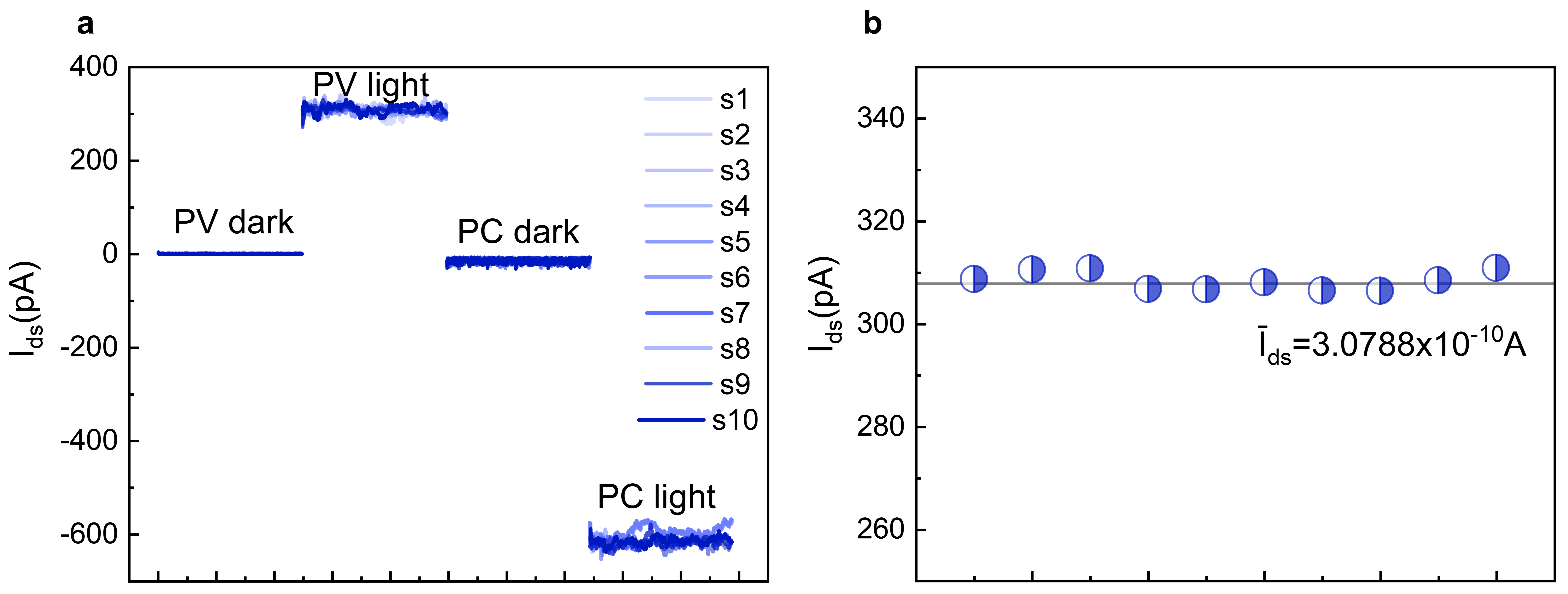


**Figure S15.** Current values of the four states in the 10-repetition test; Average current values of the PV light group.


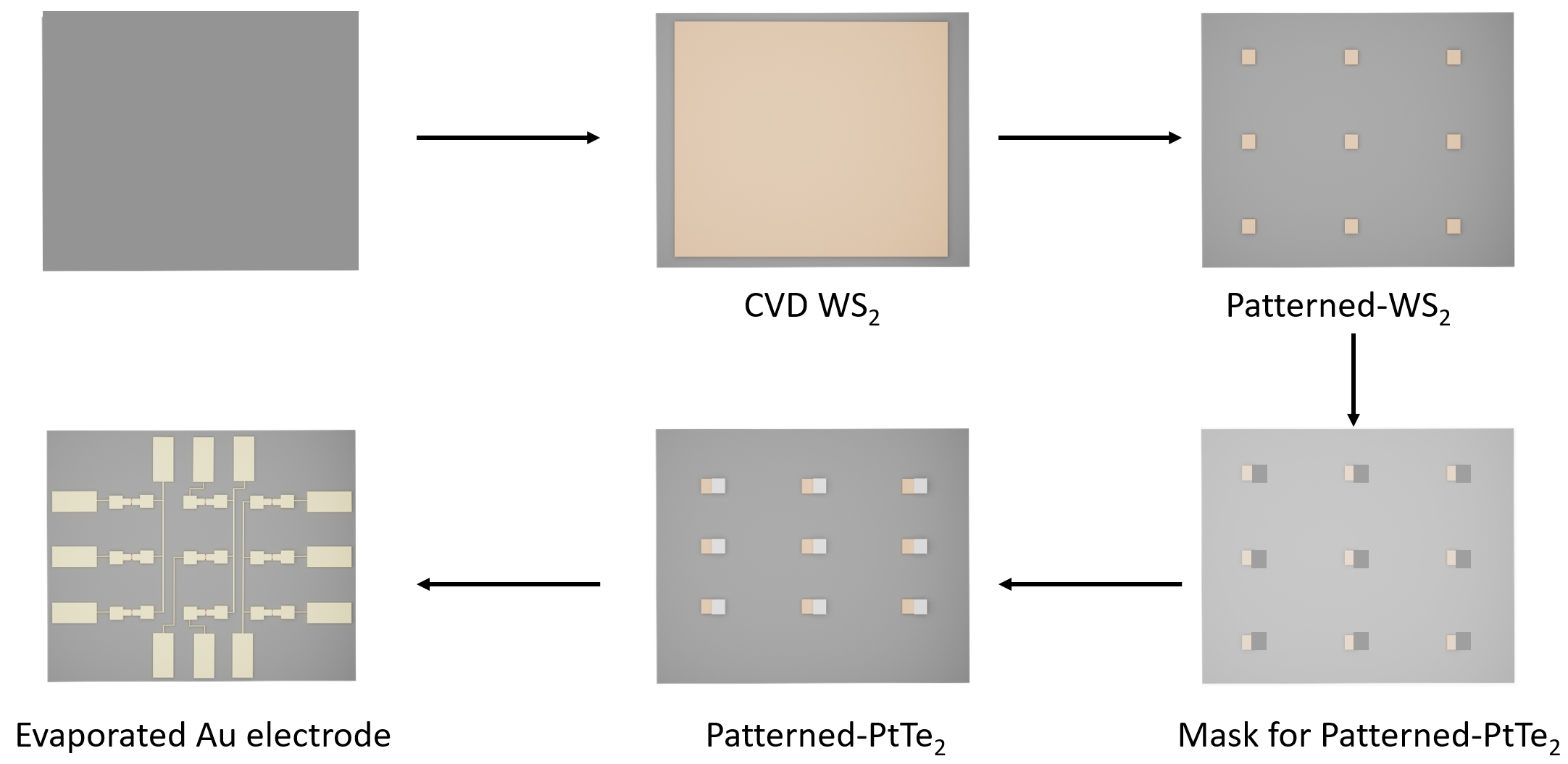


**Figure S16.** The flowchart for realizing integrated circuits.


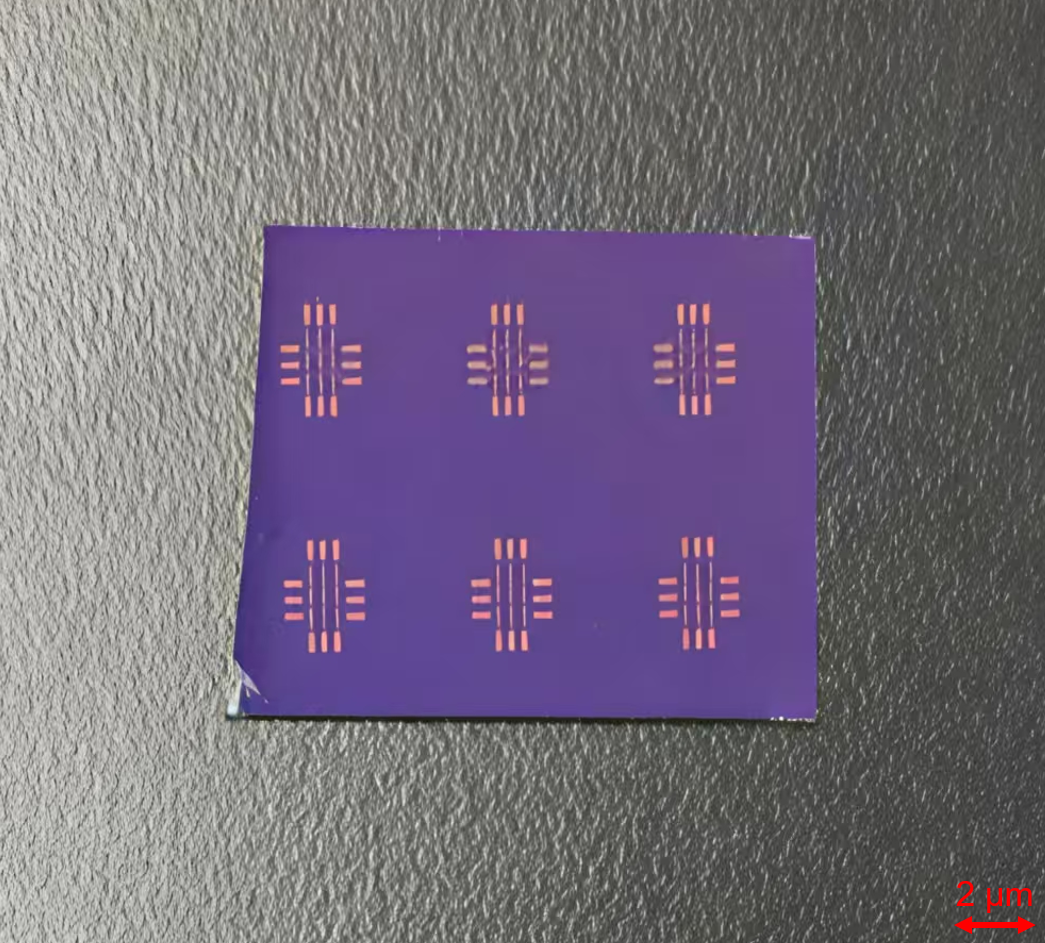


**Figure S17.** The physical image of a feasible electrode design and evaporation.

**Note1: Calculation of pixel correlation**

The pixel correlation of a four-state encrypted image is calculated as follow:^4,5^

$r_{xy}=\frac{cov\left( x,y \right)}{\sqrt{D\left( x \right)}\sqrt{D\left( y \right)}}$ (1)

$cov\left( x,y \right)=E\left[ x-E\left( x \right) \right]\left[ y-E\left( y \right) \right]$ (2)

$E\left( x \right)=\frac{1}{N}\sum_{i=1}^{N} x_{i}$ (3)

$D\left( x \right)=\frac{1}{N}\sum_{i=1}^{N} [x_{i}-E(x_{i})]^{2}$ (4)

Where *x* and *y* are the values of adjacent pixel pairs respectively (such as adjacent pixels in the horizontal direction). And *cov(x, y)*, *E(x)* and *D(x*) represent covariance, mean and variance, respectively, and N represents the total number of image pixels.

The calculation procedure first randomly selects *m* pairs (*m* < the number of matrix rows or columns) of adjacent pixels (in the horizontal, vertical, and diagonal directions). The correlation coefficients along the three directions are then calculated separately, and the final result is obtained by averaging these values.

**Note2: Calculation of the stability of the demonstration results of the device application**

To evaluate the stability of repeated measurements, we applied a small-sample t-distribution to construct confidence intervals for the population mean, supplemented by dimensionless variability (coefficient of variation, CV) and trend analysis. Specifically, we performed 10 repeated tests, calculated the mean of each measurement, and then obtained the overall mean, standard deviation, CV, and the 95% confidence interval (CI).

Taking the PV-light condition as an example:

$s=\sqrt{\frac{1}{n}\sum\left( x_{i}-\bar{x} \right)^{2}}$ (1)

$\bar{\text{x}}\text{=}\frac{\sum\text{x}_{\text{i}}}{\text{n}}\left( \text{i=1-n, n=10} \right)$ (2)

$CV=100\%\times\frac{s}{\bar{x}}$ (3)

The 95% CI for the mean is given by:

$\bar{x}\pm t_{0.9579}\frac{s}{\sqrt{n}}$ (4)

$x_{t}=\beta_{0}+t\times\beta_{1}+\varepsilon_{t}$ (5)

We further conducted a linear regression test by assigning the measurement order as t=1, 2, …,10 in the model; ε_t_ is the residual (the unobservable "true error").

The null hypothesis: H_0_: β_1_=0(no linear monotonic tendency), H_0_: β_1_≠0. Through the hypothesis testing based on probability theory, we ultimately prove that the hypothesis is valid, meaning the data are stable within the test interval.

**Table S1.** Relaxation time constants of ultrafast spectrum.

| Illumination region | V_ds_= -3 V(PC) | V_ds_= 0 V(Intrinsic) | V_ds_= 3 V(PV) |
| --- | --- | --- | --- |
| WS_2_ $\text{τ}_{\text{1}}$(ps) | 28.44 ± 2.04 | 12.36 ± 1.07 | 21.65 ± 3.12 |
| WS_2_ $\text{τ}_{\text{2}}$(ps) | 211.69 ± 1.18 | 125.26 ± 1.43 | 186.27 ± 2.49 |
| PtTe_2_/WS_2_ $\text{τ}_{\text{1}}$(ps) | 24.48 ± 2.26 | 14.06 ± 1.03 | 30.21 ± 1.49 |
| PtTe_2_/WS_2_ $\text{τ}_{\text{2}}$(ps) | 401.86 ± 2.81 | 147.26 ± 2.39 | 106.75 ± 1.45 |

**Table S2.** The article mentioned in some other literature the relevant parameters of the device.

| Devices | D^*^（Jones) | ԏ_rise_/ԏ_fall_（μs) | Ref. |
| --- | --- | --- | --- |
| GaSe/WS_2_ | 4.3×10^12^ | 37/43 | ^6^ |
| Cs_2_AgBiBr_6_/WS_2_ | 1.5×10^13^ | 52.3/53.6 | ^7^ |
| InSe/WS_2_ | 2.5×10^11^ | 63/76 | ^8^ |
| (PbS-Decorated)WS_2_ | 3.9×10^8^ | 153/226 | ^9^ |
| WSe_2_/WS_2_ | 2.3×10^10^ | 670/480 | ^10^ |
| MoS_2_/WS_2_ | 2.4×10^11^ | 9000/9000 | ^11^ |
| (WSe_2_-QDs)WS_2_ | 4.3×10^10^ | 1400/1400 | ^12^ |
| CH_3_NH_3_PbI_3_/WS_2_ | 1.0×10^12^ | 2700/7500 | ^13^ |
| SiNP array/PtTe_2_ | 2.8×10^11^ | 6.21/26.3 | ^14^ |
| Te/PtTe_2_ | 3.62×10^12^ | 7.51/36.7 | ^15^ |
| Graphene/PtTe_2_ | 2.58×10^10^ | 8.4/8.9 | ^16^ |
| PtTe_2_ | 6.92×10^9^ | 2.4/32 | ^17^ |
| PtTe_2_/MoTe_2_ | 8.2×10^11^ | 23/22 | ^18^ |
| Te/WS_2_ | 2.14×10^13^ | 10/13 | ^19^ |
| PtTe_2_/WS_2_ (PV mode) | 9.42×10^14^ | 26/22 | This work |
| PtTe_2_/WS_2_ (PC mode) | 4.68×10^12^ | 2450/139 | This work |

**Table S3.** Performance of devices with different mode-switching mechanisms.

| Article construction | D^*^（Jones) | ԏ_rise_/ԏ_fall_（μs) | Ref. |
| --- | --- | --- | --- |
| ITO/PEDOT: PSS/PBDB | 2.5×10^11^ | 40 µs/550 µs | ^20^ |
| MoTe_2_/MoS_2_ | 2.56×10^12^ | 179 ms/328 ms | ^21^ |
| ITO / Zn_x_O:D149 / PBDB-T:ITIC-Th / e-ZnO / Ag | 6.2×10^11^ | 29.52 µs/46.35 µs | ^22^ |
| hBN/bP/hBN | 8.5×10^8^ | 1.3 µs | ^23^ |
| p-WSe_2_/Gr/n-Ge | 1.2×10^12^ | 730 µs/760 µs | ^24^ |
| InSe/VO_2_ | 2.09×10^13^ | 9.7 ms/9.6 ms | ^25^ |
| WSe_2_/VO_2_ | 8.9×10^12^ | 33 ms | ^26^ |
| Ti/κ-Ga_2_O_3_/Ti | 3.51×10^13^ | 40 µs | ^27^ |
| BP/MoS_2_ | 2.35×10^11^ | 36 ms/38 ms | ^28^ |
| PbS CQD/MoS_2_/bP | 1.6×10^11^ | 20 µs | ^29^ |
| WSe_2_ / Ta_2_NiSe_5_ | 1.08×10^10^ | 91 µs | ^30^ |
| 1T′-MoTe_2_ / WSe_2_ | 4×10^10^ | 0.1 ms | ^31^ |
| PdSe_2_ / GaN | 7.9×10^12^ | 21.3 µs/78.2 µs | ^32^ |
| PtTe_2_/WS_2_ | 9.42×10^14^ | 26.3 µs/22.6 µs | This work |

**References:**

1. Zhu, Y. *et al.* Non-volatile 2D MoS2/black phosphorus heterojunction photodiodes in the near- to mid-infrared region. *Nat. Commun.* **15**, 6015 (2024).

2. Zhu, C. *et al.* Optical synaptic devices with ultra-low power consumption for neuromorphic computing. *Light Sci. Appl.* **11**, 337 (2022).

3. Wu, Q. *et al.* Waveguide-integrated twisted bilayer graphene photodetectors. *Nat. Commun.* **15**, 3688 (2024).

4. J. Hao. *et al.* A New Fractional Chaotic System and Its Application in Image Encryption With DNA Mutation. *IEEE Access* **9**, 52364-52377 (2021).

5. Ramasamy, P. *et al.* An Image Encryption Scheme Based on Block Scrambling, Modified Zigzag Transformation and Key Generation Using Enhanced Logistic-Tent Map. *Entropy Basel* **21**, 653 (2019).

6. Q. Lv. *et al.* High‐Performance, Self‐Driven Photodetector Based on Graphene Sandwiched GaSe/WS_2_ Heterojunction. *Adv.Opt. Mater.* **6**, 1700490 (2017).

7. Fang, F. *et al.* Two-Dimensional Cs_2_AgBiBr_6_/WS_2_ Heterostructure-Based Photodetector with Boosted Detectivity via Interfacial Engineering. *ACS Nano* **16**, 3985-3993 (2022).

8. Chen, J. *et al.* High-performance self-powered ultraviolet to near-infrared photodetector based on WS_2_/InSe van der Waals heterostructure. *Nano Res.* **16**, 7851-7857 (2022).

9. Yu, Y. *et al.* PbS-Decorated WS_2_ Phototransistors with Fast Response. *ACS Photonics* **4**, 950-956 (2017).

10. W. You. *et al.* Strong interfacial coupling in vertical WSe_2_/WS_2_ heterostructure for high performance photodetection. *Appl. Phys. Lett.* **120**, 181108 (2022).

11. X. Lin. *et al.* High-performance photodetector and its optoelectronic mechanism of MoS_2_/WS_2_ vertical heterostructure. *Appl. Surf. Sci.* **546**, 149074 (2021).

12. Tsai, T.-H. *et al.* Photogating WS_2_ Photodetectors Using Embedded WSe_2_ Charge Puddles. *ACS Nano* **14**, 4559–4566 (2020).

13. C. Ma. *et al.* Heterostructured WS_2_/CH_3_NH_3_PbI_3_ Photoconductors with Suppressed Dark Current and Enhanced Photodetectivity. *Adv.Mater.* **28**, 3683 (2016).

14. Tong, X.-W. *et al.* Enhancing the device performance of SiNP array/PtTe_2_ heterojunction photodetector by the light trapping effect. *Sens. Actuators Phys.* **322**, 112625 (2021).

15. Tong, X.-W. *et al.* Direct Tellurization of Pt to Synthesize 2D PtTe_2_ for High-Performance Broadband Photodetectors and NIR Image Sensors. *ACS Appl. Mater. Interfaces* **12**, 53921-53931 (2020).

16. Yu, W. *et al.* Wafer-Scale Synthesis of 2D Dirac Heterostructures for Self-Driven, Fast, Broadband Photodetectors. *ACS Nano* **16**, 12922-12929 (2022).

17. L. Zeng. *et al.* Van der Waals Epitaxial Growth of Mosaic-Like 2D Platinum Ditelluride Layers for Room-Temperature Mid-Infrared Photodetection up to 10.6 μm. *Adv. Mater.* **32**, 2004412 (2020).

18. Huang, Y. *et al.* Diverse modes regulated photoresponse and high-resolution imaging based on van der Waals semimetal PtTe_2_/semiconductor MoTe_2_ junctions. *J. Mater. Chem. C* **11**, 5045-5055 (2023).

19. Yu, H. *et al.* High-Spike Barrier Photodiodes Based on 2D Te/WS_2_ Heterostructures. *ACS Nano* **18**, 17100–17110 (2024).

20. Hu, X. *et al.* Photovoltaic and Photomultiplication Dual-Mode Near-Infrared Organic Detectors with Large Dynamic Range for Intensive and Faint Light Sensing. *Adv. Sci.* **12**, e06499 (2025).

21. Ouyang, Y. *et al.* Gate-Tunable Dual-Mode Optoelectronic Device for Self-Powered Photodetector and Optoelectronic Synapse. *Adv. Sci.* **12**, 2416259 (2025).

22 Xiao, J. *et al.* Bias-Switchable Dual-Mode Organic Photodiodes Enabled by Manipulation of Interface Layers. *Adv. Funct. Mater.* **34**, 2404711 (2024).

23. Yan, W. *et al.* Electrostatically Induced Black Phosphorus Infrared Photodiodes. *Adv. Funct. Mater.* **34**, 2316000 (2024).

24. Ding, H., Zhang, X., Zhang, L., Lin, G. & Li, C. High-performance dual-mode extended SWIR photodetector based on p-WSe_2_/graphene/n-Ge p-g-n heterostructure. *APL Mater.* **13**, 041107 (2025).

25. Wang, L. *et al.* High-Sensitivity Adjustable Operating Modes Multifunctional Detector Based on InSe/VO_2_ Heterojunction for Light and Electric Field Perception. *Adv. Opt. Mater.* **11**, 2300854 (2023).

26. Luo, H. *et al.* Phase-transition modulated, high-performance dual-mode photodetectors based on WSe_2_/VO_2_ heterojunctions. *Appl. Phys. Rev.* **6**, 041407 (2019).

27. Cui, M. *et al.* Photoconductive and photovoltaic metal-semiconductor-metal κ-Ga_2_O_3_ solar-blind detectors with high rejection ratios. *J. Phys. Appl. Phys.* **55**, 394003 (2022).

28. Lan, Z. *et al.* Dual-Band Organic Photodetectors for Dual-Channel Optical Communications. *Laser Photonics Rev.* **16**, 2100602 (2022).

29. Wang, S. *et al.* Room Temperature Bias-Selectable, Dual-Band Infrared Detectors Based on Lead Sulfide Colloidal Quantum Dots and Black Phosphorus. *ACS Nano* **17**, 11771–11782 (2023).

30. Zhu, T. *et al.* Gate Voltage- and Bias Voltage-Tunable Staggered-Gap to Broken-Gap Transition Based on WSe_2_/Ta_2_NiSe_5_ Heterostructure for Multimode Optoelectronic Logic Gate. *ACS Nano* **18**, 11462–11473 (2024).

31. Wang, H. *et al.* Polarization- and Gate-Tunable Optoelectronic Reverse in 2D Semimetal/Semiconductor Photovoltaic Heterostructure. *Adv. Mater.* **36**, 2309371 (2024).

32. Wu, D. *et al.* In Situ Fabrication of PdSe_2_/GaN Schottky Junction for Polarization-Sensitive Ultraviolet Photodetection with High Dichroic Ratio. *ACS Nano* **16**, 5545–5555 (2022).
